# Combination of HSP90 Inhibitors and HSP70 Inducers Prevent Hydrochloric Acid-Induced Pulmonary Fibrosis in Rabbits

**DOI:** 10.3390/ijms26020441

**Published:** 2025-01-07

**Authors:** Ruben M. L. Colunga Biancatelli, Pavel A. Solopov, Tierney Day, Dan E. Austin, Len E. Murray, John D. Catravas

**Affiliations:** 1 Frank Reidy Research Center for Bioelectrics, Old Dominion University, Norfolk, VA 23508, USA; psolopov@odu.edu (P.A.S.); tday@odu.edu (T.D.); lmurray@sobran-inc.com (L.E.M.); jcatrava@odu.edu (J.D.C.); 2 Division of Pulmonary and Critical Care Medicine, Department of Medicine, School of Medicine, Macon & Joan Brock Virginia Health Sciences at Old Dominion University, Norfolk, VA 23507, USA; 3 School of Medicine, Macon & Joan Brock Virginia Health Sciences at Old Dominion University, Norfolk, VA 23507, USA; austinde@evms.edu; 4 Red Hawk Veterinary Services, Poplar Branch, NC 27965, USA; 5 School of Medical Diagnostic & Translational Sciences, Ellmer College of Health Sciences, Macon & Joan Brock Virginia Health Sciences at Old Dominion University, Norfolk, VA 23507, USA

**Keywords:** Heat Shock Protein 90 (HSP90), Heat Shock Protein 70 (HSP70), geranylgeranyl acetone, TAS-116, chemically induced pulmonary fibrosis, rabbits

## Abstract

Combined therapies with Heat Shock Protein 90 (HSP90) inhibitors and Heat Shock Protein 70 (HSP70) inducers are gaining significant interest in cancer and cardiovascular research. Here, we tested the hypothesis that HSP90 inhibitors and HSP70 inducers, together, can block the development of pulmonary fibrosis. We exposed New Zealand White Rabbits to hydrochloric acid (HCl, 0.1 N, 1.5 mL/kg), one of the top five chemicals most commonly involved in accidental exposures and inhalation injuries worldwide, and treated animals with either the orally available HSP90 inhibitor TAS-116 (1.7 mg/kg 5x/week) or TAS-116 combined with the HSP70 inducer, geranylgeranyl acetone (GGA, 50 mg/kg, 3x/week). At 60 days post-HCl instillation, TAS and GGA treatment markedly reduced the degree of pulmonary fibrosis, lung dysfunction, and activation of profibrotic pathways. The use of HSP70 inducers may be a helpful tool to improve the profile of HSP90 inhibitors and reduce their minimal effective dose and side effects. Further investigation is required to explore the exact synergistic mechanism behind the antifibrotic profile of HSP90 inhibitors and HSP70 inducers.

## 1. Introduction

Heat Shock Proteins (HSPs) represent one of the most conserved and highly expressed family of chaperones [1]. Among them, HSP90 and HSP70 play a crucial role in stabilizing the tridimensional structure of numerous client proteins [2]. These proteins are also key regulators of the heat shock response. HSP70 binds to Heat Shock Factor 1 (HSF-1) in the cytoplasm, and during stress, HSF-1 detaches from HSP70, migrates into the nucleus, and enhances the expression of stress-induced genes, including HSP70 [3]. The heat shock response is initially triggered to ensure cellular and tissue homeostasis under thermal, mechanical, chemical, or inflammatory stress. However, chemical injuries can provoke a prolonged heat shock response characterized by a chronic inflammatory reaction [4,5]. This process involves two mechanisms: first, an increased production of HSPs, required to maintain protein stability during exposure to harmful chemicals, some of which can degrade and damage the proteome; second, the persistent inflammatory response necessitates elevated transcription of genes, leading to a higher demand for protein stabilization and chaperone activity. Consequently, HSP90 inhibitors have been proposed to mitigate the effects of the stress response, persistent inflammation, and chronic diseases, leading to reduced long-term injuries in affected patients [6].

Idiopathic Pulmonary Fibrosis (IPF) is a chronic and incurable disease characterized by the aberrant deposition of extracellular matrix proteins, causing progressive scarring of the lungs, a reduction of air spaces, and respiratory failure, with poor outcomes, often fatal within 3–5 years from diagnosis [7]. Occupational exposures to dust (wood, silica, asbestos fibers), metals, cigarette smoke, infections (flu, Epstein–Barr virus, hepatitis, herpes), and chemical fumes increase the risk of developing IPF [8,9]. Additionally, it is estimated that many patients with occupational exposures are misdiagnosed with IPF due to the incomplete collection of environmental and occupational exposure histories [10]. Currently, pirfenidone and nintedanib are the only FDA-approved therapies for IPF. They slow the decline in forced vital capacity (FVC), but do not improve survival [11,12]. Thus, investigating novel therapeutic approaches to block or even reverse the fibrotic process in the lungs remains a significant but as-yet-unfulfilled goal. While bleomycin-induced pulmonary fibrosis is considered the standard animal model for IPF, other chemicals have also shown reproducible results in inducing stable chronic lung lesions [13].

Hydrochloric acid (HCl), widely used in various industries and in laboratory research, is transported in quantities of 20 million metric tons annually. HCl inhalation leads to the development of chronic injuries, such as reactive airway dysfunction syndrome (RADS) [14], asthma-like conditions [15,16,17], and even pulmonary fibrosis [18]. Steel workers exposed to HCl experienced persistent respiratory symptoms, including cough, shortness of breath, and wheezing, and exhibited reduced FVC and FEV1, even at concentrations as low as 2 ppm, previously considered safe by the American Conference of Governmental Industrial Hygienists. Upon inhalation, HCl dissolves in airway fluids, penetrating tissues and provoking a persistent inflammatory response [19]. In animal models of HCl exposure, mice exhibit a strong initial inflammatory response that subsides by day 4, followed by a mild residual activation of profibrotic pathways lasting over 30 days, culminating in an overall picture of chemically induced pulmonary fibrosis [20,21]. Preliminary reports in mice suggest that this prolonged inflammatory response is mediated by transforming growth factor-beta (TGF-β), the activation of type 2 macrophages, and injury to alveolar and endothelial barriers [15,22,23,24,25]. Our previous work demonstrated that HSP90 inhibitors are beneficial at various levels, blocking the TGF-β/Raf/RAS/MAPK axis, Smads translocation, reducing extracellular matrix (ECM) production, and preventing endothelial and epithelial barrier dysfunction, thereby successfully mitigating chemically induced chronic lung dysfunction [24,26,27,28]. We have defined optimal doses, maximal delayed administration, and minimal effective treatment, and also demonstrated that the levels of HSP70 influence the HSP90 therapeutic profile [29].

In a recent study, we characterized the pulmonary toxicity of HCl in New Zealand White Rabbits, identifying histological, functional, and molecular markers of disease, notably at 60 days post-exposure [30]. Rabbits have been pivotal in preclinical research and are essential for the further investigation of new drugs and their translation to clinical trials [31]. Rabbit respiratory and gastrointestinal systems are highly sensitive and offer a complex immune response [32]. The fibrosis observed in rabbits after HCl exposure closely resembles the type of injury seen in IPF patients, with fibroblast activation foci, honeycombing, and reduced air spaces. In this study, we investigated whether an HSP90 inhibitor (TAS-116: 1.7 mg/kg, 5x/week orally) administered alone or together with an HSP70 inducer (geranylgeranyl acetone (GGA): 50 mg/kg, 3x/week orally) can block the HCl-induced fibrotic response and development of chronic lung injury in New Zealand White Rabbits.

## 2. Results

### 2.1. TAS-116 and GGA Ameliorate HCl-Induced Inflammation in Bronchoalveolar Lavage Fluid

**Figure 1:**
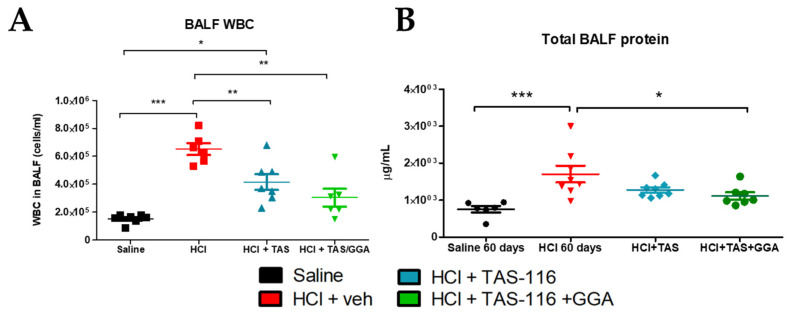
TAS-116 and GGA improve HCl-induced alveolar inflammation. Rabbits instilled with HCl or saline were treated with vehicle (30% corn oil), TAS-116 (1.7 mg/kg 5x/week per os), or TAS-116 and GGA (50 mg/kg 3x/week per os) between days 4 and 21 post HCl. Bronchoalveolar lavage (BAL) fluid was analyzed for (**A**) cellularity and (**B**) total protein content. n = 5–6; *: *p* < 0.05; **: *p* < 0.01; ***: *p* < 0.001 with 1-way ANOVA and Tukey’s post hoc test.

New Zealand White Rabbits of approximately 1 kg body weight were intratracheally instilled with HCl 0.1 N or saline (1.5 mL/kg) and after 4 days began treatments with vehicle (corn oil), TAS-116 (1.7 mg/kg, 5x/week per os), or TAS-116 and GGA (50 mg/kg, 3x/week per os). Treatments were continued until day 21 and the animals were euthanized at day 60 post HCl instillation. Bronchoalveolar lavage fluid (BALF) was analyzed for cellularity and proteinosis. HCl produced a robust increase in alveolar cellularity and protein concentration (Figure 1). Rabbits treated with TAS-116 alone showed a reduction in BALF white blood cells, which was further improved when TAS-116 was combined with GGA (Figure 1A). At 60 days post HCl, the amount of total protein in the BALF was increased in rabbits receiving HCl and vehicle, but statistically unchanged compared to saline-instilled controls in both HCl + TAS- and HCl + TAS/GGA-treated rabbits (Figure 1B).

### 2.2. TAS-116 and GGA Ameliorate the HCl-Induced Activation of Pro-Fibrotic Markers

**Figure 2:**
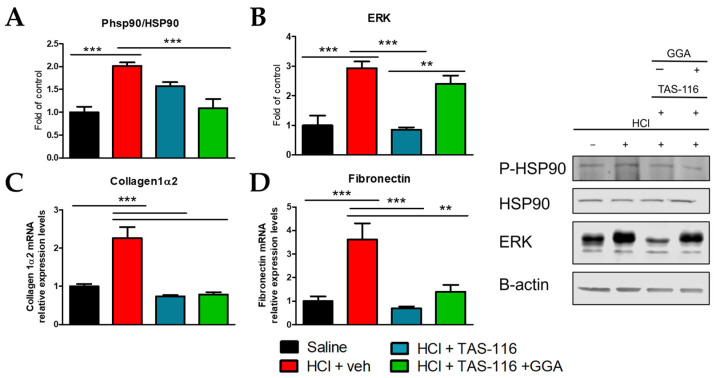
TAS-116 alone and in combination with GGA modulates HCl-stimulated profibrotic pathways. Rabbits instilled with HCl or saline were treated with vehicle (30% corn oil), TAS-116 (1.7 mg/kg 5x/week per os), or TAS-116 and GGA (50 mg/kg 3x/week per os) between days 4 and 21 post HCl. Proteins were extracted from lung homogenates and analyzed by WB for (**A**) P-HSP90/HSP90 and (**B**) ERK. RNA was extracted from tissues and analyzed by RT-PCR for (**C**) collagen1α2 and (**D**) fibronectin. Β-actin was used as housekeeping to normalize data, and data were plotted as a fold of controls. N = 4–6; **: *p* < 0.01; ***: *p* < 0.001 with 1-way ANOVA and Tukey’s post hoc test.

We have recently shown that HCl instillation in rabbits provokes a profibrotic response mediated by HSP90 and macrophage type 2 via the overexpression of CD163 [30]. Treatment with TAS-116 alone or in combination with GGA reduced the HCl-induced activation of HSP90, i.e., phosphorylation, but only the TAS/GGA combination was able to lower its activation to the levels of controls (Figure 2A). ERK was similarly increased in HCl-instilled rabbits, and only treatment with TAS-116 was able to block the HCl-induced constitutively expression of ERK (Figure 2B). However, both therapeutic approaches were able to significantly reduce the HCl-increased expression levels of collagen 1α2 and fibronectin, analyzed by RT-PCR (Figure 2C,D).

### 2.3. TAS-116 and GGA Prevent the Development of Pulmonary Fibrosis

**Figure 3:**
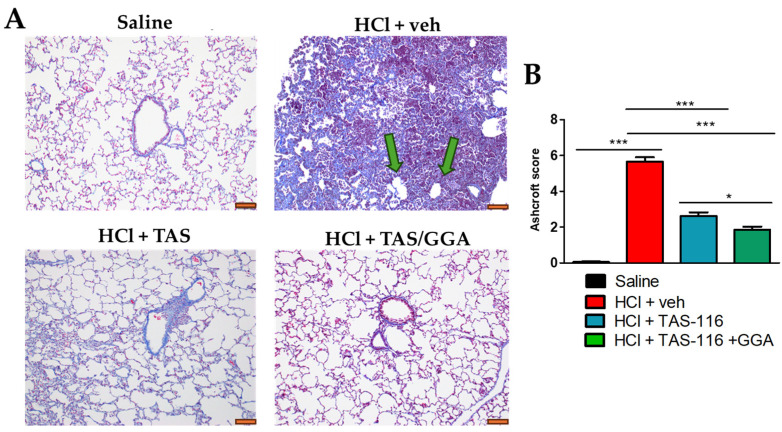
TAS-116 and GGA improve HCl-induced pulmonary fibrosis. Rabbits instilled with HCl or saline were treated with vehicle (veh: 30% Corn oil), TAS-116 (1.7 mg/kg 5x/week per os), or TAS-116 and GGA (50 mg/kg 3x/week per os) between days 4 and 21. At day 60, mice were euthanized and the lungs were harvested, fixed in 10% formaldehyde, and stained for Masson’s trichrome (**A**). The green arrows indicate honeycombing areas in the HCl-instilled group not present in the other groups. Chronic lung injury was quantified via the Ashcroft score (**B**). Original magnification 20×, orange scale bar 100 µm. N = 5–6; *: *p* < 0.05; ***: *p* < 0.001 with 1-way ANOVA and Tukey’s post hoc test.

The HCl-exposed rabbits displayed a complete loss of lung architecture. The alveolar structures were replaced by a dense scar, positive to collagen staining for Masson’s trichrome. Furthermore, we observed empty circular structures, compatible with the clinical definition of honeycombs, a typical feature of IPF [33] (green arrow, Figure 3A). Rabbits instilled with HCl and treated with TAS or TAS/GGA showed a milder level of chronic lung injury, minimal fibrotic response, and a conserved alveolar and septal lung structure (Figure 3A). As quantified by the Ashcroft score, the TAS/GGA group had the greatest improvement when compared to HCl-instilled rabbits treated with vehicle or even with TAS-116 alone (Figure 3B).

### 2.4. TAS-116 and GGA Improve HCl-Induced Lung Dysfunction

**Figure 4:**
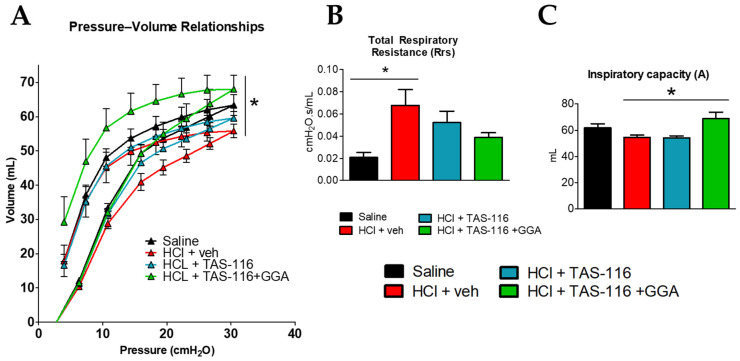
TAS-116 and GGA improve HCl-induced lung dysfunction. Rabbits instilled with HCl or saline were treated with vehicle (30% corn oil), TAS-116 (1.7 mg/kg 5x/week per os), or TAS-116 and GGA (50 mg/kg 3x/week per os) between days 4 and 21. Lung function studies, performed at day 60, collected data for (**A**) pressure–volume relationships (PV loops), (**B**) respiratory system resistance (Rrs), and (**C**) inspiratory capacity (A). n = 5–6; *: *p* < 0.05 with 1-way ANOVA and Tukey’s post hoc test.

The anesthetized rabbits were connected to a Flexivent respirator (SCIREQ), equipped with module 6 for medium-sized animals, 60 days after instillation with HCl or saline. The HCl-instilled rabbits showed a downward shift in the pressure–volume (PV) relationships (Figure 4A). The lower PV loop indicates that lungs reach maximal intrapulmonary pressures at lower volumes, and it is a sign of reduced FVC. The TAS-treated rabbits exhibited improved PV loop relationships, but only rabbits in the TAS/GGA group regained the normal PV loops similar to controls (Figure 4A). HCl also elicited an increase in respiratory system resistance (Rrs), indicative of a “stiff” lung. TAS/GGA was able to prevent such an increase and maintain values similar to saline-instilled rabbits (Figure 4B). The TAS/GGA rabbits displayed higher inspiratory capacity (A) than all other groups (Figure 4C).

## 3. Discussion

HCl exposure provokes acute lung injury through direct chemical damage to the alveolar and endothelial epithelium followed by a strong inflammatory immune response [34,35]. Previous studies in rabbits have demonstrated the presence of pulmonary edema and significant frothy fluid accumulation in the bronchi one hour post HCl instillation, predominantly affecting the lower cranial lobe and the peripheral and basal regions of the caudal lobes [36]. Despite initial heterogeneous inflammation, our current findings reveal a diffuse pulmonary fibrosis phenotype at 60 days, indicating that initial localized inflammation transitions into a more systemic profibrotic response. This is corroborated by Masson’s trichrome staining, which shows extensive collagen expression, and a high Ashcroft score with low standard error (Figure 3B).

To inhibit the fibrotic process while not interfering with the initial pro-inflammatory response, we initiated treatment with HSP90 inhibitors, either alone or in combination with HSP70 inducers, on day 4 when inflammation began to subside, and ceased treatments on day 21 to avoid disrupting normal lung healing [27]. It is well known that, in IPF, different pro-fibrotic pathways are activated via fibroblast activation, oxidative stress, persistent inflammatory response, epithelial and endothelial mesenchymal transformation, deposition of a fibrotic scar, and a progressive reduction in FVC leading to respiratory failure [37,38]. Decaris et al. demonstrated striking increases in fibrillar collagen synthesis in mice that occur 1 to 3 weeks post bleomycin exposure, consistent with a pathogenic accumulation of ECM [39]. We observed a similar time response in HCl- and NM-challenged mice, with increased levels of interstitial, peribronchial, and perivascular collagen deposition [20,22,40]. Notably, HSP90 inhibitors (AUY-922, TAS-116, or AT13387) effectively prevented HCl-induced pulmonary fibrosis in mice [24,27,41]. Our group has also recently tested the combination of TAS-116 with GGA, obtaining promising results in mice [29]. Here, the combination of TAS-116 with GGA, the HSP70 inducer, was similarly associated with a much lower degree of pulmonary fibrosis, the maintenance of normal alveolar/septal architecture, and a lack of honeycomb areas, implying the reduced production of mucus and exudates in the lungs [42]. This is further confirmed by the fact that rabbits receiving TAS-116 and TAS/GGA treatments display much lower levels of ECM proteins, such as collagens and fibronectin, and blocked activation of HSP90 (Figure 2). Interestingly, TAS/GGA-treated rabbits showed persistent constitutive (total) expression of ERK, unlike those treated with TAS alone. ERK, a kinase involved in multiple extracellular signaling pathways including TGF-β [43], plays a major role in pulmonary fibrosis, with MEK/ERK inhibition being beneficial in blocking its progression [44]. We have previously shown that HSP90 inhibitors and combined HSP90 inhibitors and HSP70 inducers inhibit ERK activation (phosphorylation) in mice [29]. The persistent increase in total ERK, without evidence of its activated, i.e., phosphorylated, status is not necessarily an indicator of increased TGF-β signaling. Indeed, this could imply that HSP90 inhibition by TAS destabilizes ERK proteins, potentially triggering a negative feedback mechanism promoting ERK de novo gene expression and transcription, explaining the observed increase in total ERK levels [45]. ERK belongs to the non-canonical TGF-β signaling (Ras/RAF/MAPK/ERK/MEK), which is fully stabilized by HSP90 [46], and its inactivation may have affected multiple regulatory mechanisms. Conversely, in other studies on cancer cell lines, HSP70 pharmacologic or genetic blockade resulted in ERK inhibition [47], suggesting that these two proteins may have a similar positive correlation, and thus justifying our observed increase in total ERK levels during the overexpression of HSP70 by GGA.

In this study, we utilized TAS-116, an inhibitor of HSP90α and HSP90β isoforms, with minimal effects on other isoforms such as GRP4 and TRAP-1 [48,49]. HSP90 exists in several isoforms, e.g., HSP90α, HSP90β, and mitochondrial HSP90, emerging over 500 million years ago due to gene duplication [50]. These isoforms assemble in dimers (HSP90αα, HSP90ββ, HSP90αβ) assuming a V-shape conformation with the ATP binding site in the N-domain where client proteins are assisted during folding and protein maturation [51]. Homologous or heterologous dimers (HSP90αα, HSP90ββ, HSP90αβ) display different binding properties [52], with HSP90β demonstrating specific microtubule interaction properties [53,54], while HSP90α is linked to caspase-3 and STAT1 [52,55]. Sontake et al. showed that the inhibition of the HSP90αβ isoform disrupts ECM production and fibroblast migration and proliferation, while the loss of HSP90αα does not, indicating the coordinated activation of the two isoforms in fibrogenesis [56].

Here, treatment with TAS-116 was combined with GGA to stimulate the effects of HSP70. Indeed, reduced levels of HSP70 have been found in fibroblasts isolated from IPF patients and IPF lungs, making HSP70 inducers a promising additional therapy for pulmonary fibrosis [57,58]. Rabbits receiving combined therapy displayed the highest pressure–volume (PV) loops, indicating favorable lung mechanics and reduced HCl-induced respiratory resistance (Rrs), a major indicator of lung stiffness and fibrosis (Figure 4). PV loops are indeed a translatable measurement that correlates to the FVC measured clinically [59]. Over recent years, HSP90 inhibitors have been associated with HSP70 inducers or HSP70 inhibitors to boost their therapeutic profile. HSP70-HSP90 simultaneous inhibition provokes increased oxidative stress, the activation of autophagic pathways, and even the modulation of the transcriptome {Sable, 2018 #141}. In cancer, this double inhibition has been associated with stronger effects on cancer cell death, as the expression of HSP70 is considered a protective mechanism of cells to survive treatments with HSP90 inhibitors {Kudryavtsev, 2017 #142}. In our work, however, we employed much lower doses of HSP90 inhibitors to avoid cellular death and instead modulate proliferative pathways, and added an HSP70 inducer, which protects cells from the injury induced by HCl. This inhibitor–inducer combination enhanced the therapeutic effects, potentially through increased antioxidant activity, cellular cytoprotection, and inhibition of apoptotic pathways [57]. Importantly, we carefully computed the dose of TAS-116 and GGA from our previous work conducted in mice, as shown in Section 4.4, to guarantee the replicative effects across species and the future clinical translatability of this therapeutic approach.

Our study has limitations. The lack of rabbit-specific antibodies hindered our understanding of fibrotic pathways during HCl exposure and treatment with HSP90 inhibitors and HSP70 inducers, limiting the mechanistic depth of our work. Additionally, we only utilized a single dose for both TAS and GGA, warranting further studies to optimize dosing and timing. Furthermore, additional studies are required to understand the exact role of each of the HSP90 isoforms in the fibrotic process in order to develop inhibitors with higher therapeutic profiles and fewer side effects. Nevertheless, our study provides the first evidence of the beneficial effects of HSP90 inhibition and HSP70 induction in chemically induced pulmonary fibrosis in a non-rodent species, laying the groundwork for the further development of this class of drugs into clinical applications.

## 4. Materials and Methods

### 4.1. Materials

Hydrochloric acid (37%), ACS grade; methacholine chloride, USP grade; radioimmunoprecipitation assay (RIPA) buffer; and protease inhibitor cocktail were supplied by Sigma-Aldrich Corporation (St. Louis, MO, USA). AnaSed (xylazine), USP grade; Ketaset (ketamine), USP grade; and buprenorphine were obtained from Covetrus (Portland, ME, USA). Formaldehyde ACS reagent, 37%, was purchased from ThermoFisher Scientific (Waltham, MA, USA); the BCA Protein Assay Kit from Pierce Co. (Rockford, IL, USA); and EDTA from GE Healthcare (Chicago, IL, USA). TAS-116 was purchased from MedChem Express (Monmouth Junction, NJ, USA) and geranylgeranyl acetone from Sigma Aldrich (Milwaukee, WI, USA). ERK, P-HSP90, and HSP90 antibodies were obtained from ThermoFisher Scientific (Waltham, MA, USA), EDTA and Western blot membranes from GE Healthcare (Chicago, IL, USA), TRIzol and SuperScript VILO reverse transcriptase kit from Invitrogen (Carlsbad, CA, USA), RNeasy Mini Kit from Qiagen (Hilden, Germany), and SYBR Green Master Mix from Applied Biosystems (Carlsbad, CA, USA). All primers used for real-time quantitative PCR were purchased from Integrated DNA Technologies, Inc. (Coralville, IA, USA). For SDS-PAGE, ProtoGel (30% acrylamide mix) and TEMED were from National Diagnostics (Atlanta, GA, USA), Tris–HCl buffer from Teknova (Hollister, CA, USA), 10% SDS and ammonium persulfate from Thermo Fisher Scientific, and Protein Dual Color Standards and Tricine Sample Buffer from Bio-Rad Laboratories (Hercules, CA, USA). All antibodies were purchased from a reputable commercial source and have published immunospecificity data.

### 4.2. Ethical Statement

Animal studies were approved by the Institutional Animal Care and Use Committee (IACUC) of Old Dominion University (Protocol #23-013 approved on 06/02/2020) and abide by the principles of animal experimentation, as published by the American Physiological Society.

### 4.3. Animals and Treatment Groups

Male New Zealand White Rabbits (Albino strain #052) weighing 1.0–2.5 kg were purchased from Charles River Laboratories. The animals were anesthetized intramuscularly with ketamine (20–50 mg/kg) and xylazine (3–5 mg/kg). The animals were then injected with buprenorphine (0.12 mg/kg s.c.) and 10–12 mL/kg of sterile saline to provide analgesia and prevent dehydration, respectively. After confirming adequate anesthesia through the absence of pain reflexes, the animal was placed in dorsal recumbency. The neck was shaved and washed twice with alcohol and 1.5 L/min of oxygen was provided via a face mask. Then, a 25 G catheter needle was inserted between the tracheal rings, the needle removed, and the catheter advanced for ~1 inch. Proper localization of the catheter within the tracheal lumen was confirmed by aspiring air without feeling resistance. Finally, saline (1.5 mL/kg) or 0.1 N HCl (1.5 mL/kg) was rapidly injected through the catheter and flushed with 4 mL/kg of air. The catheter and needle were then removed, the rabbit moved to the upright position, and the rabbit placed in the sternal position. The oxygen flow was progressively weaned by 0.5 l/min every 10 min to finally room air. During the procedures, the animal’s heartrate, temperature and SpO_2_ were continuously monitored. The animals were then observed for an additional 30 min before being returned to their cages and then monitored hourly for the first day.

The rabbits were randomly divided into four treatment groups:

(1) Rabbits instilled with normal saline (Saline);

(2) Rabbits instilled with 0.1 N HCl 1.5 mL/kg and treated with vehicle (HCl + veh);

(3) Rabbits instilled with 0.1 N HCl 1.5 mL/kg and treated with TAS-116 (1.7 mg/kg per os 5x/week);

(4) Rabbits instilled with 0.1 N HCl 1.5 mL/kg and treated with TAS-116 and GGA (1.7 mg/kg per os 5x/week + 50 mg/kg 3x/week per os).

In all groups, treatment started at day 4 and ended by day 21 post HCl instillation, as published [29]. The animals were monitored daily. At the end of the experimental period (day 60), the animals were sedated with Acepromazine (1.5 mg/kg i.m.), ketamine (40 mg/kg i.m.), and xylazine (5 mg/kg i.m.) and connected to an animal respirator (Flexivent, SCIREQ). At the end of the respiratory studies, the animals received a lethal dose of pentobarbital (120 mg/kg i.v.) in the marginal ear vein. To complete all of the proposed experiments, a total of 73 rabbits were employed.

### 4.4. TAS-116 and GGA Rabbit Equivalent Dose

There are no standardized conversion charts for translating drug therapeutics between species. In our previous studies on mice [1], doses of 7 mg/kg TAS-116 and 200 mg/kg GGA successfully prevented the development of pulmonary fibrosis. To adapt these doses for rabbits, we first converted the doses from mice to humans and then from humans to rabbits using body surface area (BSA) calculations, as follows [2]:

1. For TAS-116, we calculated the human equivalent dose (HED) using a mouse-to-human conversion factor of 12.3.

$$TAS-116\;HED=\frac{7\;mg/kg}{12.3}=0.57\;mg/kg\;(HED)$$

2. Next, we computed the rabbit animal equivalent dose (AED) from the HED using a human-to-rabbit conversion factor of 3.1.

TAS−116 Rabbit AED=0.57 mg/kg×3.1=1.76 mg/kg (HED)

For GGA, we followed the same procedure, starting with a dose of 200 mg/kg, to calculate the HED and then the AED:

$$GGA\;Rabbit\;AED=\frac{200\;mg/kg}{12.3}\times 3.1=50.4\;mg/kg\;(HED)$$

Thus, we established starting doses of TAS-116 1.7 mg/kg and GGA 50 mg/kg.

### 4.5. Bronchoalveolar Lavage Fluid (BALF)

To collect BALF, we injected and then retrieved 10 mL of sterile 1x PBS through a syringe cannula connected with 1000 µL micropipette tip. This device perfectly fits the diameter of the rabbit’s trachea without losing any of the PBS in the extrapulmonary space. A hemocytometer was used to count the white blood cells (WBCs). Then, following centrifugation of the fluid at 2500× *g* for 10 min, the supernatant was harvested to measure the protein levels. The concentration of total protein was assessed using a micro bicinchoninic acid (BCA) Protein Assay Kit, adhering to the guidelines provided by the supplier.

### 4.6. Histopathology, Immunohistochemistry, and Lung Fibrosis Scoring

The rabbits were euthanized with 120 mg/kg pentobarbital i.v. in the marginal ear vein, their chests were opened, and the lungs were fixed in 10% formaldehyde. Mid-transverse sections were cut from the formalin-fixed lung tissues and then embedded in paraffin. Then, 5 µm thick sections from these blocks were stained with Masson’s trichrome. Twenty randomly selected fields from each slide were examined at 20× and 40× magnifications. An investigator, unaware of the group identities, used the Ashcroft scoring methods to evaluate the degree of pulmonary fibrosis [60,61].

### 4.7. Lung Tissue Collection

Immediately after euthanasia, the lungs were flushed by injecting saline and EDTA in the pulmonary artery. The lungs were then dissected from the thorax, cut into pieces and snap-frozen for later analyses.

### 4.8. Western Blot Analysis

Proteins in the lung tissue homogenates were extracted from frozen lungs by sonication (50% amplitude, three times for 10 s) in ice-cold RIPA buffer with added protease inhibitor cocktail (100:1). The protein lysates were gently mixed for 3 h at 4 °C, centrifuged twice at 14,000× *g* for 10 min. Equal amounts of protein, were mixed with Tricine Sample Buffer 1:1, boiled for 5 min, and then separated on a 10–12% polyacrylamide SDS gel by electrophoresis, transferred to a nitrocellulose membrane, incubated with the appropriate primary antibody, followed by incubation with the secondary antibody, and then detected by digital fluorescence imaging (LI-COR Odyssey CLx, Dallas, TX, USA). Beta-actin was used as the loading control. ImageJ software v.1.8.0 was used to perform densitometric quantification of the bands (http://imagej.nih.gov/ij; National Institutes of Health, Bethesda, MD, USA, accessed on 10 November 2024).

### 4.9. Lung Mechanics Measurements

The rabbits were anesthetized as described in paragraph 4.3. When a lack of pain reflexes was observed, a longitudinal neck incision of ~1 inch was made in the neck region to expose the trachea. Then, a single horizontal cut of 1/6 inch was made between the tracheal rings and a plastic 1000 µL micropipette tip was inserted and connected to a Flexivent small animal ventilator equipped with FX6 module for medium-sized animals (SCIREQ Inc., Montreal, QC, Canada). Ventilation was performed at a tidal volume of 10 mL/kg and a respiratory rate of 150/min. A 15 min stabilization period was allowed before measurements began. Initially, pressure–volume (PV) loops were assessed by incrementally increasing the airway pressure to 30 cm H_2_O and then decreasing it, reflecting the lungs’ intrinsic elasticity. Subsequently, Snapshot-150 and Quick Prime-3 maneuvers were executed. Respiratory system resistance (Rrs) and inspiratory capacity (A) measurements were averaged over 12 recordings. At the end of the respiratory studies, the rabbits were euthanized with 120 mg/kg pentobarbital i.v.

### 4.10. RNA Isolation and Quantitative Real-Time PCR (qPCR)

Lung tissue, stored in an RNAlater solution, was dried and homogenized in TRIzol^®^, followed by a cleaning-up step using the RNeasy Mini Kit. The purified RNA was transcribed into cDNA using the SuperScriptTM IV VILO Reverse Transcriptase Kit and was analyzed by real-time qPCR with SYBR Green Master Mix on a StepOne Plus Real-Time PCR System (Applied Biosystems v.2.3). The results were evaluated using the standard curve method and were expressed as the fold of the control values, normalized to β-actin. Specifically designed primer pairs and qPCR conditions were applied to selectively determine the expression of rabbit β-actin and collagen 1α2.

### 4.11. Statistical Analysis

Data are shown as means ± standard error of the mean. We assessed the statistical significance of differences between groups using one- or two-way analysis of variance (ANOVA), accompanied by Tukey’s or Bonferroni’s post hoc tests. Statistical evaluations were conducted using GraphPad Prism v. 9.0 (GraphPad Software, San Diego, CA, USA). A *p*-value of less than 0.05 indicated significant differences between groups.

## 5. Conclusions

HSP90 inhibitors are promising candidates to block the fibrotic process in the lungs. Coadministration with an HSP70 inducer shows promise in stabilizing the effects of TAS-116, providing stronger preclinical data in rabbits. Further investigation of this class of drugs may provide valid clinical candidates for patients with occupational pulmonary fibrosis.

## Data Availability

Data can be made available by the authors upon reasonable request.
